# Application of Fibrin-Laminin Hydrogel Concurrent with Electrically Stimulated Eccentric Training Hinders Recovery in Volumetric Muscle Loss

**DOI:** 10.3390/jfb17020102

**Published:** 2026-02-19

**Authors:** Natalia Ziemkiewicz, Jeffrey Au, Hannah Chauvin, Preston Shake, Manvee Vuppala, Koyal Garg

**Affiliations:** School of Science and Engineering, Saint Louis University, Saint Louis, MO 63103, USA; natalia.ziemkiewicz@gmail.com (N.Z.); manvee.vuppala@slu.edu (M.V.)

**Keywords:** skeletal muscle, hydrogels, rehabilitation

## Abstract

Regenerative rehabilitation can enhance skeletal muscle recovery following trauma-induced volumetric muscle loss (VML). We previously optimized fibrin-laminin hydrogels for muscle regeneration and an electrically stimulated eccentric contraction training (EST) for muscle rehabilitation. The goal of this study was to examine the combined effect of these two therapies on maximizing tissue recovery. A VML defect was created by removing ~20% of muscle mass from the tibialis anterior (TA) muscle in adult male Lewis rats. The injured TA muscles were treated with fibrin-laminin (FBN450) hydrogel. EST was implemented 2 weeks post-injury at both 100 Hz and 150 Hz frequencies and continued for 4 weeks. The results showed no improvement in muscle mass or function with combined FBN450 and EST application. Histological analysis revealed significantly reduced type 2B myofiber cross-sectional area (CSA) and percentage in the combined hydrogel and EST treatment group. Gene expression studies showed >20-fold higher inflammatory (e.g., CCR7, CD163) and fibrotic (e.g., Col1a1) signaling, with no concomitant increase in myogenic markers in the hydrogel + EST group. Collectively, these results indicate that the FBN450 hydrogel therapy did not synergize with EST to improve outcomes following VML.

## 1. Introduction

Orthopedic trauma is frequently characterized by volumetric muscle loss (VML), defined as the surgical or traumatic excision of muscle tissue (>20%) resulting in chronic functional deficits [1]. Skeletal muscles commonly sustain mild or moderate injury and have remarkable regenerative capabilities; however, in VML, the tissue architecture is disrupted, impairing regenerative capacity. The etiology of VML is the loss of muscle fibers and native elements, such as satellite cells and extracellular matrix (ECM) components, leading to permanent loss of contractile tissue. Consequently, due to the characteristics of VML, such as prolonged inflammation and persistent strength deficits, patients often experience disfigurement and/or chronic disability [1,2,3]. Clinical options for reconstructing or repairing muscle tissue following VML are extremely limited. As a result, VML contributes to permanent disability in civilian and military patients [2].

In previous studies, fibrin-laminin hydrogels were developed for VML treatment [4,5,6]. Implantation of LM-111 (450 μg/mL)-enriched fibrin (FBN450) hydrogels supported myogenic protein expression, increased the ratio of contractile tissue to fibrotic tissue, increased the quantity of neuromuscular junctions, and resulted in a higher quantity of small to medium-sized myofibers (500–2000 μm^2^). Overall, the FBN450 hydrogel enhanced functional muscle regeneration, improving muscle force by ~60% compared to the untreated VML group at 28 days post-injury. Collectively, these results showed that an acellular therapy, such as the FBN450 hydrogels, can provide a promising therapeutic treatment for VML.

To improve regeneration and function following trauma, the synergistic application of both regenerative and rehabilitation strategies is being explored [7,8,9,10,11,12,13]. In a previous study, we optimized an electrical-stimulation eccentric-contraction training (EST) program by implementing the regimen 14 days post-VML injury for 4 weeks. Briefly, the training program consisted of altering the mechanical load on VML-injured muscles by modulating the stimulation frequency (50 Hz, 100 Hz, & 150 Hz) for a total of 20 eccentric contractions per bout. Our results show that the implementation of the electrical stimulation protocol at both 100 Hz and 150 Hz improved muscle mass (~37% and ~39%, respectively) and mean myofiber CSA. However, muscle function was only improved with 150 Hz stimulation (by ~34%) compared to a non-trained VML sham group [14]. In this study, we chose to utilize both frequencies to better understand the stimulation threshold at which rehabilitation leads to functional recovery.

We hypothesized that the regenerative therapy (FBN450 hydrogel) would support myofiber hyperplasia, while the rehabilitation technique (EST) would concurrently drive hypertrophy of these regenerated fibers. This combinatorial approach was expected to yield superior muscle mass and strength. Therefore, the objective of this study was to determine the extent of functional repair and regeneration following the combined application of FBN450 and EST in a rodent VML model.

## 2. Materials and Methods

### 2.1. Hydrogel Synthesis

The hydrogels were synthesized by combining murine Laminin-111 (LM-111, 6 mg/mL, Trevigen, Gaithersburg, MD, USA), fibrinogen from bovine plasma (20 mg/mL, MilliporeSigma, St. Louis, MO, USA), thrombin from bovine plasma (20 U/mL, MilliporeSigma), a protease inhibitor (PI) cocktail (1:1000, MilliporeSigma), and calcium chloride (20 mM, MilliporeSigma). These LM-111-enriched fibrin hydrogels were synthesized with a final concentration of 450 μm/mL LM-111 (FBN450), as previously described [4,5,6]. Briefly, fibrinogen and a PI cocktail were added to a warmed isotonic sodium chloride solution. LM-111 was added to the fibrinogen solution. In a 6-well plate, fibrinogen solution was added, followed by calcium chloride and, lastly, thrombin. Once all components were added, the well plate was incubated for 1 h at 37 °C to allow complete gelation.

### 2.2. Rodent Model of VML

All animal work was conducted in strict adherence with the Animal Welfare Act, the Animal Welfare Regulations, and the principles of the Guide for the Care and Use of Laboratory Animals. The experimental protocol was reviewed and approved by the Institutional Animal Care and Use Committee at Saint Louis University (IACUC protocol# 2645). Adult (10–12 weeks old) male Lewis rats (Charles River Laboratories, Wilmington, MA, USA) were housed in a vivarium accredited by the Association for Assessment and Accreditation of Laboratory Animal Care International and provided with food and water *ad libitum*. All animals were in good health and were allowed a one-week acclimation period prior to experiments.

All surgical procedures were performed under continuous isoflurane anesthesia (1.5–3.5%) with body temperature maintained, as described previously [4,15]. A unilateral VML injury was created in the TA muscle of the right hind limb. Roughly ~20% of the muscle mass was removed from the center using a 6 mm biopsy punch to create a full-thickness VML defect. The removed muscle mass was weighed for consistency. Fibrin hydrogels with an LM-111 concentration of 450 μm/mL (FBN450) were implanted to completely fill the defect site (~10 mm × 0.5 mm). The fascia was closed using coated Vicryl (5-0, Ethicon, Somerville, NJ, USA) sutures in a simple, interrupted manner to secure the implanted hydrogel, followed by closing the wound with skin staples and Prolene (5-0, Ethicon) sutures in a simple, interrupted manner. Pain management was provided at the time of surgery through a single use of sustained-release buprenorphine (1 mg/kg), which was administered subcutaneously (s.c.) at the nape of the neck. A total of 16 animals were randomly divided into three experimental groups. The FBN450 sham group (control group) received no electrical-stimulated eccentric training (EST), while the FBN450 + 100 Hz and FBN450 + 150 Hz groups received EST at 100 Hz and 150 Hz, respectively.

Post-operative body weights were monitored daily for 72 h; a transient weight loss of approximately 5–10% was observed, followed by a gradual recovery. Following a 42-day experimental period, animals were euthanized via thoracotomy and exsanguination under deep anesthesia. The tibialis anterior (TA) muscles (n = 5–6 per group) were excised, weighed, and processed for subsequent histological and biomolecular analyses. All animals were included in the analysis. Investigators were blinded to group allocation, and the animals were identified by an alphanumeric code.

### 2.3. Electrical Stimulation Protocol

**Figure 1:**
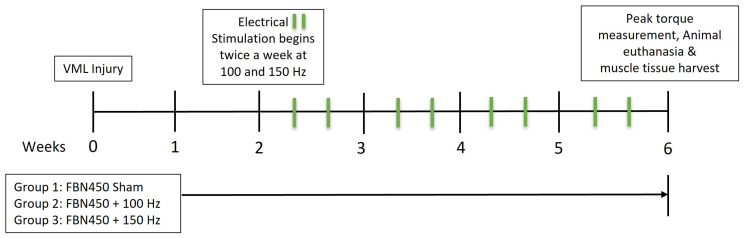
Schematic representation of the experimental design.

In this work, myofibers were tetanically activated by peroneal nerve stimulation during lengthening. This ensured mechanical loading across active cross-bridges, which is essential for adaptive remodeling and hypertrophy [16]. EST of the injured leg began 2 weeks post-injury and continued for 4 weeks, as shown in the experimental design (Figure 1). To maximize the potential for adaptive remodeling, the EST was deferred until two weeks after hydrogel implantation. This delay was introduced to ensure a baseline level of structural repair at the VML-injured site, resolve acute inflammation, and facilitate hydrogel-tissue integration [14]. Briefly, the right foot of the animal was secured to an Aurora Scientific servomotor (Model 305C; Aurora Scientific Inc., Aurora, ON, Canada) with adhesive tape. The peroneal nerve was stimulated via percutaneous platinum electrodes. Optimal stimulation parameters (30–35 mA) were established to evoke maximal isometric twitch contractions, utilizing a 0.2 ms pulse width and a 0.3 s train duration at 1 Hz. Twenty eccentric contractions were performed by stretching the anterior crural muscles from 19° of ankle dorsiflexion to 19° of ankle plantarflexion, while simultaneously stimulating the peroneal nerve with the optimal current at 100 or 150 Hz [14]. Each rat performed 4 sets of 5 eccentric contractions twice weekly for a total of 8 sessions. Ten seconds of rest were given between contractions, and 2 min of rest were given between sets. The EST protocol was designed to minimize exercise-induced muscle damage and fatigue. Repetition ranges and rest periods were optimized through pilot testing to facilitate recovery between EST sessions. A sham group, with a unilateral VML injury treated with an FBN450 hydrogel, was used to determine the effects of anesthesia and electrode placement. For the sham group, the knee was clamped and the electrodes were placed. However, the sham group neither received isometric twitch contractions nor underwent the EST protocol.

### 2.4. In Vivo Functional Testing

Peak isometric torque was measured to assess functional recovery of the anterior crural muscles 42 days post-injury, as previously described [4,17]. Briefly, rats were placed on a heated platform with their right foot secured to the foot pedal on the servomotor shaft (Model 305b, Aurora Scientific). Two platinum electrodes were inserted subcutaneously on either side of the peroneal branch of the sciatic nerve. Optimal current (30–35 mA) was set to elicit maximal isometric twitch contractions (train frequency 1 Hz, pulse width 0.2 ms, duration 0.3 s). A force-frequency curve was generated by eliciting isometric contractions at 20, 60, 100, 150, and 200 Hz, with 1 min rest intervals.

### 2.5. Histological and Immunohistochemical Analysis

For histological analysis, the TA muscle was divided axially at the defect midpoint. The top portion was frozen in 2-methylbutane (Fisher Scientific, Hampton, NH, USA) in a liquid nitrogen bath. Muscle cross-sections (15 µm) were stained with hematoxylin and eosin (H&E) to assess tissue morphology. Imaging was performed using a Zeiss Axiocam fluorescent microscope or slide scanners such as Olympus BX614S (Saint Louis University, St. Louis, MO, USA) or NanoZoomer 2.0 HT (Washington University in Saint Louis, St. Louis, MO, USA).

TA muscles (n = 5/group) from each animal were cryosectioned and stained with the Picrosirius Red (PSR) Stain kit (Polysciences, Catalog no. 24901). The muscle samples were fixed in 10% formalin, rinsed with DI water, and stained with PSR for 60 min. Then, the samples were rinsed in HCl solution, rinsed again with DI water, and dehydrated in 70% ethanol for 30 s. A glass cover slip was secured over the samples with Cryoseal™. The samples were then imaged using polarized light microscopy with TerziCam Pro software (Terzic Instruments LLC; Ballwin, MO, USA), and the area fraction of total collagen, as well as collagen fractions stained red, yellow, and green, were determined using a custom MATLAB code (R2024b).

Muscle sections were stained using antibodies from the Developmental Studies Hybridoma Bank (DSHB, Iowa City, IA, USA) for myofiber types 1 (1:20; BA.D5), 2A (1:50; SC.71), and 2B (1:20; BF.F3). A laminin (1:100; ab11575) antibody was used to identify myofiber outlines. Unstained fibers were identified as type 2X. The MyoQuant MATLAB program [18] for image analysis was used to quantify the myofiber cross-sectional area (CSA) and fiber-type distribution (n = 5–6 muscles/group). In the FBN450 sham group, 5623 ± 435.3 (mean ± standard error of mean) fibers were detected and analyzed in the MyoQuant MATLAB program (R2024b). In the FBN450 + 100 Hz and FBN450 + 150 Hz groups, 3628 ± 327.2 fibers and 5713 ± 804.5 fibers were detected and analyzed, respectively.

### 2.6. Gene Expression Analysis

TA muscle (n = 5–6/group) segments of approximately 50 mg containing the defect area and surrounding musculature were homogenized in Trizol LS reagent, followed by RNA isolation and purification using the RNAeasy mini kit (Qiagen, Hilden, Germany). The RNA yield was quantified using the NanoDrop 2000c Spectrophotometer (Thermo Scientific, Waltham, MA, USA), and the 260/280 and 230/260 ratios were used to verify purity. RNA was reverse-transcribed using iScript Reverse Transcriptase Supermix (Bio-Rad, Hercules, CA, USA). Primer sets were synthesized by the Millipore Sigma oligos design tool (Table 1). Aliquots (2 μL) of cDNA were amplified in triplicate with 300 nM forward/reverse primers using SYBR GreenER (Invitrogen, Carlsbad, CA, USA) on a Bio-Rad CFX96 thermal cycler (Bio-Rad). Gene expression was normalized to 18S (reference gene) to determine the ΔCT value. Expression levels for each mRNA transcript were determined by the 2^−ΔΔCT^ method by normalizing each group to uninjured contralateral control muscles.

### 2.7. Statistical Analysis

GraphPad Prism 9 was used for all statistical analyses and sample sizes were decided based on previous studies [4,14]. Data are presented as the mean ± standard error of the mean (SEM). An unpaired *t*-test and ordinary one-way analysis of variance (ANOVA, or two-way ANOVA (treatment × time)) were used when appropriate to determine if there was a significant interaction or main effect between variables. The treatment groups included FBN450 sham, FBN450 + 100 Hz, and FBN450 + 150 Hz. A Fisher’s least significant difference *post hoc* comparison was performed to identify significance, with *p*  <  0.05.

## 3. Results

### 3.1. Muscle Mass

**Figure 2:**
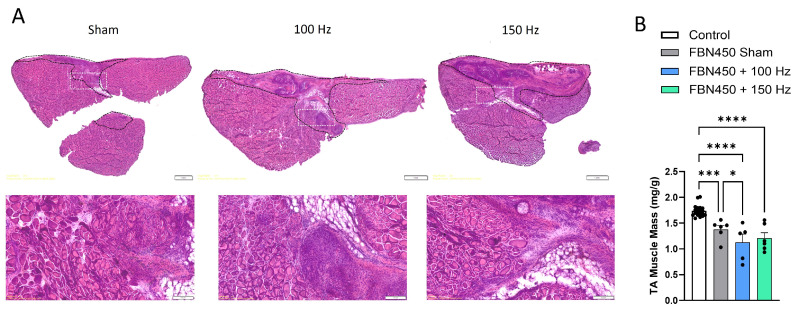
(**A**) Muscle sections were stained with H&E at 42 days post-injury. Dashed yellow lines approximate the defect region and the white boxes represent the magnified regions in the panel below. (**B**) The muscle mass of the VML-injured TA muscles was measured 42 days post-injury (n = 5–6 muscles/group). Data Analysis was performed using two-way ANOVA. “*” indicates a significant difference (* *p* < 0.05, *** *p* < 0.001, **** *p* < 0.0001) between groups.

Muscle sections stained with H&E (Figure 2A) show increased cellular infiltration into the VML defect in the EST groups relative to the hydrogel-alone group. Qualitatively, upon removal of the muscles during the time of sacrifice, muscles in the FBN450 + 100 Hz group and FBN450 + 150 Hz group appeared to have an indented groove at the site of the VML injury. TA muscle mass (Figure 2B), normalized to body weight, at 42 days post-injury, showed significant differences between the uninjured contralateral control muscles and all VML injured muscles, irrespective of treatment (ANOVA, *p* < 0.0001). Relative to the uninjured control muscles, deficits of ~21%, ~35%, and ~30% were observed in the FBN450 sham, FBN450 + 100 Hz, and FBN450 + 150 Hz groups, respectively. Additionally, the FBN450 Sham group had significantly greater muscle mass than the FBN450 + 100 Hz group (*p* = 0.0446).

### 3.2. Eccentric Torque and Muscle Strength

**Figure 3:**
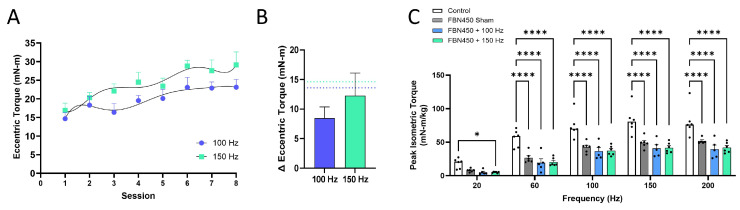
(**A**) Eccentric torque was higher with stimulation at 150 Hz and increased with each bout. (**B**) The Δ eccentric torque between bout 8 and bout 1 is reported, showing no differences between the 100 Hz and 150 Hz programs when a hydrogel was introduced. The blue and green dotted lines represent the Δ eccentric torque for untreated VML-injured muscles following EST training for 4 weeks from Ziemkiewicz et al., JOR 2023 [14]. Data analysis was performed using an unpaired *t*-test. (**C**) Peak isometric torque production was measured and raw data were collected and normalized to body weight. Data analysis was performed using two-way ANOVA. “*” indicates a significant difference (* *p* < 0.05, **** *p* < 0.0001) between groups.

Eccentric torque of the muscle was recorded during each exercise session or bout (Figure 3A). A progressive increase in eccentric torque was observed with the implementation of the 100 Hz and 150 Hz EST programs following FBN450 hydrogel implantation. Overall, eccentric torque at 150 Hz was higher than that recorded at 100 Hz (Two-way ANOVA; Interaction, *p* =0.9713; Time Factor, *p* < 0.0001; Frequency Factor, *p* = 0.0003). The change in average eccentric torque (Figure 3B; Unpaired *t*-test, *p* = 0.4332) between bout eight and bout one was 8.462 Hz and 12.24 Hz for the 100 Hz and 150 Hz training programs, respectively.

Functional recovery of the muscle was assessed by measuring peak isometric torque production at 6 weeks post-injury, after implantation of an FBN450 hydrogel and implementation of EST for four weeks. Peak isometric torque produced at different frequencies (20–200 Hz) of stimulation (Figure 3C) was normalized to the animal’s body weight. The results showed that torque at 20 Hz and 60 Hz was lower than torque at all other frequencies (Interaction, *p* = 0.1739; Stimulation factor, *p* < 0.0001; Treatment factor, *p* < 0.0001). The uninjured control muscles produced significantly higher torque than all VML-injured groups, irrespective of treatment. At 200 Hz, the deficits in torque relative to the uninjured control muscles were ~36%, ~51%, and ~48% in the FBN450 sham, FBN450 + 100 Hz, and FBN450 + 150 Hz groups, respectively.

In previous studies, the peak isometric torque measured at 150 Hz was 51.9 ± 19.1 N-mm/kg with EST alone [14] and 56.2 ± 5.2 N-mm/kg with biosponge + EST treatment [7]. In contrast, peak isometric torque was 41.7 ± 7.8 N-mm/kg with the FBN450 + EST treatment, showing a ~20–26% lower value compared to previous studies. The increase in eccentric torque was 14.6 N-mm with EST alone, 18.6 N-mm with biosponge + EST treatment, but only 12.23 N-mm with FBN450 treatment, indicating a 16–34% drop in eccentric torque. Taken together, this comparative analysis suggests that the FBN450 hydrogels did not synergize with EST and consistently underperformed across various metrics, failing to reach even the baseline levels of untreated VML-injured tissue.

### 3.3. Myofiber Cross-Sectional Area

**Figure 4:**
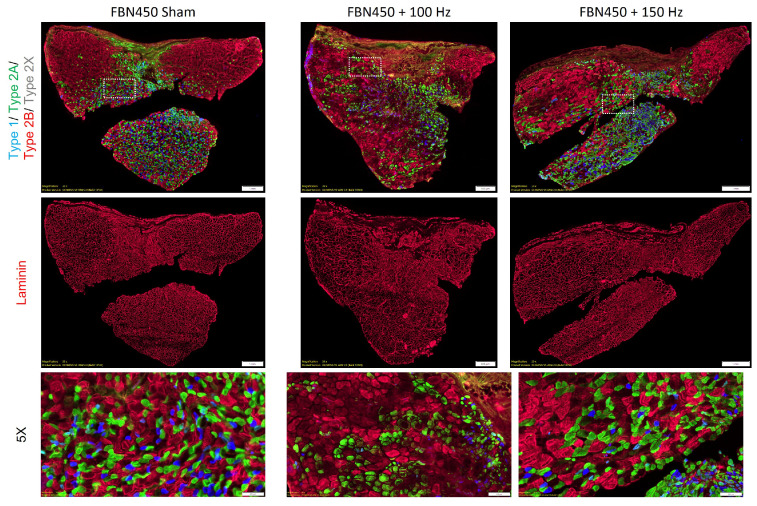
Muscle sections were stained for various fiber types and laminin (n = 5–6 muscles/group) at day 42 post-injury.

**Figure 5:**
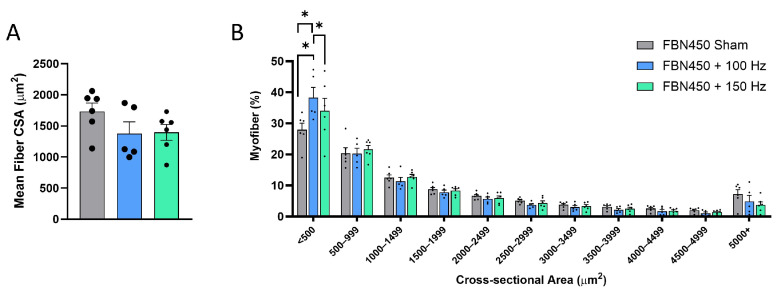
The mean fiber cross-sectional area (CSA) (**A**) and global fiber size distribution (**B**) were quantified using a custom-designed image analysis MATLAB program. The CSA distribution analysis showed that myofibers in the size range of <500 μm^2^ were increased in the FBN450-treated muscles following electrical stimulation training at 100 Hz and 150 Hz compared to the FBN450 Sham muscles. Data analysis for the mean fiber CSA was performed by an ordinary one-way ANOVA. Data analysis for the fiber size distribution was performed using a two-way ANOVA. Significance between groups is indicated by * (*p* < 0.05). “*” indicates a significant difference (* *p* < 0.05) between groups.

Muscle sections stained for specific myofiber types and outlined with laminin were used to quantify the myofiber count and cross-sectional area (CSA) and determine the number of slow-twitch versus fast-twitch myofibers (Figure 4). The mean myofiber CSA was statistically similar (One-way ANOVA, *p* = 0.1967) between treatment groups (Figure 5A). For uninjured control muscles, the mean CSA was reported to be 2216.181 ± 376.685 in our previous study at day 42 post-injury [14]. Therefore, deficits in mean CSA relative to control muscles were ~23%, ~38%, and ~36% for the FBN450 sham, FBN450 + 100 Hz, and FBN450 + 150 Hz groups, respectively. The myofiber CSA distribution analysis (Figure 5B; Interaction, *p* = 0.0112) revealed a smaller percentage of myofibers in the FBN450 sham group for the <500 µm^2^ size when compared to both the FBN450 + 100 Hz (*p* < 0.0001) and FBN450 + 150 Hz (*p* = 0.0007) groups. The FBN450 + 100 Hz group also had a greater percentage of myofibers in the <500 mm^2^ size range when compared to the FBN450 + 150 Hz (*p* = 0.0227) group.

**Figure 6:**
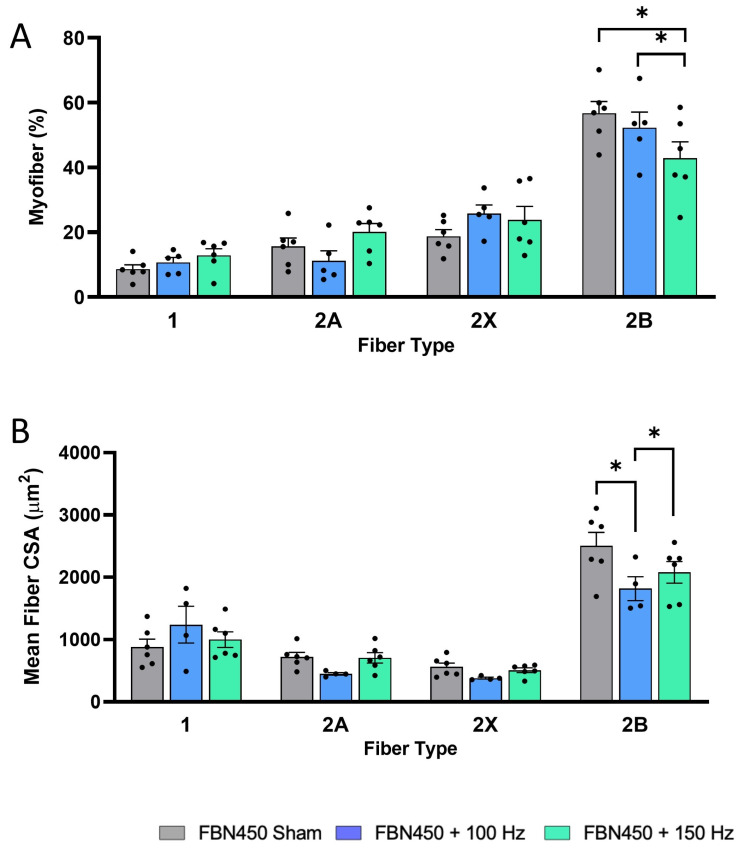
The percentage and mean fiber CSA of 2B fibers increased in the FBN450 Sham group. (**A**) The percentage of fiber type-specific fibers in each treatment group and the (**B**) fiber type-specific mean fiber CSA were determined using a custom-designed image analysis MATLAB program. Data analysis was performed using a two-way ANOVA. “*” indicates a significant difference (* *p* < 0.05) between groups.

**Figure 7:**
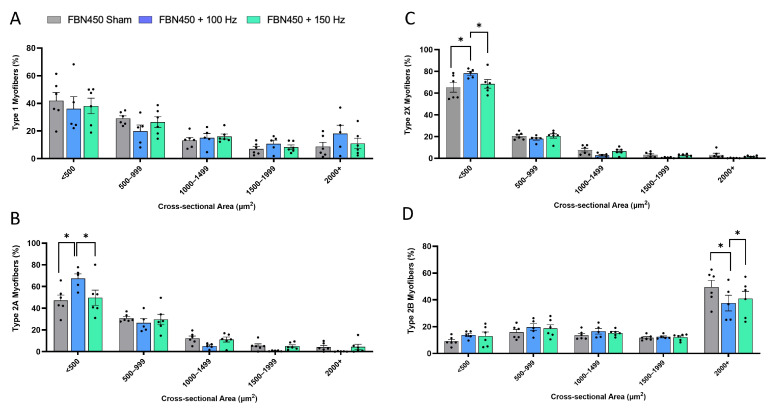
Fiber size distributions for (**A**) type 1, (**B**) type 2A, (**C**) type 2B, and (**D**) type 2X were quantified using the MyoQuant MATLAB program. Data analysis was performed using a two-way ANOVA. “*” indicates a significant difference (* *p* < 0.05) between groups.

Fiber type-specific CSA analysis revealed a decrease in the percentage of fast-twitch glycolytic type 2B myofibers in the FBN450 + 150 Hz group (Figure 6A; Interaction, *p* = 0.0140) when compared to the FBN450 sham group (*p* = 0.0024) and FBN450 + 100 Hz group (*p* = 0.0447). The CSA analysis revealed no changes in the percentage of either type 1, type 2A, or type 2X myofibers, irrespective of treatment. The mean CSA (Interaction, *p* = 0.0401) of type 2B myofibers was also significantly lower in the electrically stimulated muscles (FBN450 + 100 Hz (*p* = 0.0015) and FBN450 + 150 Hz (*p* = 0.0235) groups) compared to the FBN450 sham group (Figure 6B). Fiber type distribution analysis showed no differences in type 1 myofibers (Figure 7A). However, the FBN450 + 100 Hz group had a greater percentage of both small-diameter (<500 µm^2^) slow-twitch oxidative type 2A myofibers (Interaction, *p* = 0.0028; Figure 7B) and fast-twitch glycolytic type 2X myofibers (Interaction, *p* = 0.0030; Figure 7C) compared to the FBN450 sham group and FBN450 + 150 Hz group. The FBN450 sham group had a significantly higher percentage of large (>2000 µm^2^) myofibers than both the FBN450 + 100 Hz and FBN450 + 150 Hz groups (Figure 7D).

### 3.4. Collagen Deposition and Gene Expression Analysis

**Figure 8:**
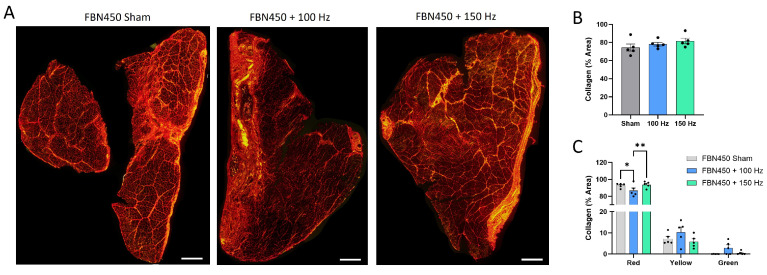
Muscle cross-sections were stained with (**A**) Picrosirius Red (PSR) and imaged using polarized microscopy. (**B**) Total collagen area fraction was determined using thresholding by a custom MATLAB program. (**C**) Total area fraction of collagen separated into red, yellow, and green regions was quantified. “*” indicates a significant difference (*p < 0.05, ** *p* < 0.01) between groups.

Muscle cross-sections stained with PSR and visualized under polarized light (Figure 8A) revealed that total collagen remained similar between groups (Figure 8B; One-way ANOVA, *p* = 0.2956). There was a significant reduction in densely packed collagen fibers (red) [19] within the FBN450 + 100 Hz group compared to both the FBN450 sham and FBN450 + 150 Hz groups (Figure 8C; Interaction, *p* = 0.0082; packing, *p* < 0.0001). However, no significant differences were observed in the deposition of intermediate (yellow) or immature/loosely packed (green) collagen fibers across any treatment groups (*p* > 0.9999).

**Figure 9:**
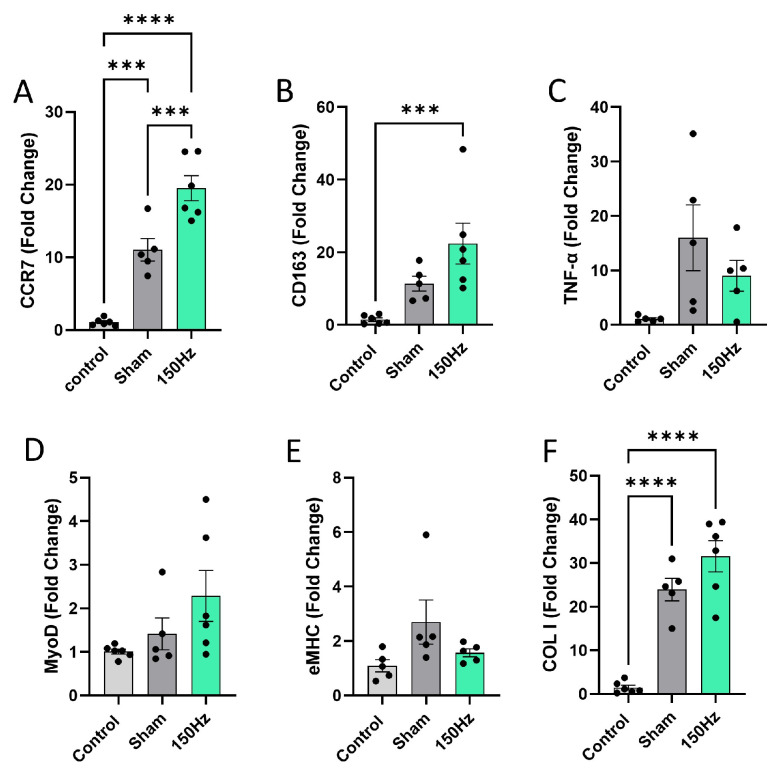
Gene expression analysis of (**A**) proinflammatory marker CCR7, (**B**) anti-inflammatory marker CD163, (**C**) inflammatory cytokine TNF-α, (**D**) myoblast marker MyoD, (**E**) embryonic myosin heavy chain (eMHC), and (**F**) collagen type 1 (COL1) was performed 6 weeks post-injury. Data analysis was performed using a two-way analysis of variance (ANOVA). “*” indicates a significant difference (*** *p* < 0.001, **** *p* < 0.0001) between groups.

Due to a lack of differences between the 100 Hz and 150 Hz groups in terms of muscle mass, function, and mean CSA, gene expression analysis was performed to investigate the biomolecular response of FBN450-treated skeletal muscle to EST at 150 Hz only. Results showed significantly increased expression of CCR7 (Figure 9A), which was an M1 macrophage phenotype-associated marker in both the FBN450 hydrogel-treated sham group and the combined FBN450 + 150 Hz EST group. The application of EST further increased CCR7 expression (One-way ANOVA, *p* ≤ 0.0001). The gene expression of CD163 (Figure 9B; One-way ANOVA, *p* = 0.0033), an M2 macrophage-associated marker, was only elevated in the FBN450 + 150 Hz EST group. Although the TNF-α expression was 16-fold and 9-fold higher in the FBN450 hydrogel-treated sham group and the FBN450 + 150 Hz-treated EST group, statistically significant differences were not observed (Figure 9C; One-way ANOVA, *p* = 0.0545). Both an early-stage myogenic marker, MyoD (Figure 9D; One-way ANOVA, *p* = 0.1000), and a late-stage myogenic marker, eMHC (Figure 9E; One-way ANOVA, *p* = 0.1031), showed no differences between groups. The gene expression of collagen 1 (Figure 9F; One-way ANOVA, *p* < 0.0001) was found to be significantly elevated in both FBN450 (~24 fold) and FBN450 + 150 Hz EST (~32 fold) groups.

## 4. Discussion

The objective of this study was to investigate the efficacy of combining a regenerative therapy, FBN450 hydrogel, with a rehabilitation program, EST, for functional repair of VML in a rodent model. Although FBN450 [4] and EST [14] have previously demonstrated improved regeneration and function when used individually, the combined application of these therapies in this study unexpectedly hindered muscle recovery.

The lack of synergistic improvement in the hydrogel plus EST group, despite the well-documented benefits of EST in untreated [14] and biosponge-treated [7] VML models, suggests a fundamental breakdown in the regenerative rehabilitation paradigm. In previous studies, we observed a lack of integration between the hydrogel and the surrounding muscle tissue at days 7 and 14 post-injury [4]. Therefore, by remaining physically separated from the surrounding musculature, the hydrogel likely created a structural discontinuity that might have prevented the seamless transfer of mechanical cues. This is most clearly evidenced by the significant decrease in the percentage of Type 2B myofibers and the cross-sectional area (CSA). Type 2B fibers are highly glycolytic and the most sensitive to mechanical unloading [20]. These fibers have fewer mitochondria and rely on consistent mechanical loading to maintain their large CSA and protein synthesis. Therefore, their atrophy following the combined application of hydrogel and EST suggests that the hydrogel shielded the surrounding musculature from mechanical stress. Rather than the muscle experiencing the mechanical loading of eccentric exercise, the hydrogel may have absorbed or dampened these forces, effectively keeping the surrounding musculature (especially Type 2B myofibers) in a state of functional disuse.

This mechanical shielding appears to have fundamentally altered the cellular response to rehabilitation. While hydrogel and EST can separately and individually drive myogenesis [4,14], their combined application led to a significant elevation in Collagen 1 gene expression without increased myogenic gene expression. Interestingly, while Collagen 1 gene expression was upregulated, total collagen protein levels remained similar across groups. This suggests a state of high-turnover remodeling where fibrotic signaling is accelerated, but potentially coupled with an equal rate of degradation, resulting in a disorganized extracellular matrix rather than functional tissue bridging.

The immunological profile of the hydrogel + EST group further explains this regenerative failure. The simultaneous elevation of M1-like (i.e., CCR7) and M2-like (i.e., CD163) macrophage markers suggests both a heightened and stalled inflammatory response. In a healthy healing trajectory, a clear transition from a pro-inflammatory M1 phase to a pro-regenerative M2 phase is required [21,22]. The persistent presence of both markers indicates a chronic foreign-body response, likely exacerbated by the hydrogel’s mechanical motion during EST. We speculate that interfacial micromotion between the non-integrated hydrogel and the host tissue led to recurrent micro-trauma, which further drove pro-inflammatory signaling. This persistent inflammatory signaling is known to be antagonistic to myogenesis and can actively drive myofiber atrophy. In this study, we used gene expression to determine whether the hydrogel + EST therapy provided sufficient stimulus to trigger a molecular repair response. While we acknowledge that mRNA levels do not always correlate with protein abundance, this transcriptomic approach enabled us to determine cellular responsiveness to EST. Collectively, our data suggests elevated inflammatory and fibrotic signaling coupled with a lack of a myogenic response. These results identify molecular barriers that future studies can validate and address to improve recovery outcomes.

Ultimately, these results reveal a selective synergy between biomaterials and eccentric exercise. While the biosponge scaffolds used in previous studies likely allowed for better load-sharing and cellular infiltration [7], the FBN450 hydrogel lacked the mechanical and structural features necessary for a synergistic effect with EST. Our findings demonstrate that synergy is not an inherent or guaranteed property of combining a scaffold with exercise. Instead, it is an emergent outcome dependent on the biomaterial’s mechanical and physical properties with the rehabilitative stimulus. If the biomaterial fails to integrate or share the mechanical load, as seen with the FBN450 hydrogel, the relationship between the scaffold and exercise can become antagonistic, leading to activation of inflammatory and fibrotic pathways. This highlights a critical need to engineer biomaterials that are specifically tuned to synchronize with rehabilitation. In fact, many studies have successfully combined biomaterials with various rehabilitation approaches in both humans and animal models [12].

Furthermore, we speculate that the timing of the intervention likely turned the eccentric training regimen into a secondary insult. At 14 days post-injury, the hydrogel was only partially present at the defect site [4]. We posit that the intense EST program may have accelerated the remodeling and degradation of the biomaterial before functional tissue bridging could occur. Consequently, the defect site became a void, leaving the injured muscle vulnerable to an intense training regimen that aggravated an already traumatic and chronic injury. In contrast, the FBN450 sham group, undisturbed by premature mechanical loading via EST, had ample time to elicit its regenerative capabilities, ultimately resulting in improved function. These findings highlight the necessity of a synchronized approach to recovery, where the intensity and timing of rehabilitation are calibrated to the structural integrity and biological stage of the implanted scaffold.

The current study is not without limitations, particularly regarding the intervention’s fixed temporal window. While we hypothesized that delaying the EST by 2 weeks would represent a viable therapeutic window, the lack of real-time monitoring of the hydrogel’s structural integrity remains a constraint. Future research should use non-invasive imaging modalities (such as ultrasound) to track the hydrogel’s degradation kinetics and host-tissue integration longitudinally [23]. This would allow for a more objective determination of when the scaffold has successfully transitioned from a passive filler to an integrated, load-bearing conduit capable of withstanding the mechanical stresses of EST. Another limitation of this study is the exclusive use of male rats. This design was chosen to minimize the physiological variability inherent in mixed sex studies. However, recognizing that sex is a critical biological variable, these studies provide the necessary framework for future investigations in female rodents to elucidate sex-specific differences in recovery outcomes.

Building upon the findings of this study, several future directions are warranted to optimize the selective synergy between the FBN450 hydrogel and EST. A critical next step is to investigate whether the electrical stimulation protocol should be delayed, perhaps beginning at 21 days post-injury rather than 14 days. This extended undisturbed period might provide the regenerative therapy ample opportunity to fully integrate with the remaining musculature and establish a robust cellular niche before being subjected to mechanical strain due to EST. By allowing the hydrogel to elicit its full regenerative capacity and stabilize the defect site, a later introduction of EST may yield the functional improvements that were absent in the current study.

Conversely, while an earlier introduction of the training protocol could, in theory, provide more exercise sessions and lead to greater strength gains, previous studies suggest that this approach should be handled with caution [24,25]. Starting EST too soon after injury may inadvertently accelerate remodeling and premature hydrogel degradation, thereby creating a void at the defect site during a critical phase of myogenesis. This highlights a complex trade-off in regenerative rehabilitation, where the muscle requires early mechanical stimulus to prevent atrophy, but the biomaterial requires stability to facilitate repair. Future studies should therefore focus on identifying the goldilocks zone, an optimal time window in which the mechanical properties of the scaffold are sufficient to support load and the biological state of the muscle is primed to receive a hypertrophic stimulus provided by exercise.

Collectively, our findings reveal that synergy in regenerative rehabilitation is a conditional phenomenon, dependent on a match between the biomaterial’s properties and the intensity and timing of the rehabilitative stimulus. Successful recovery requires a synchronized approach to activate myogenesis rather than fibrotic and inflammatory cascades.

## Figures and Tables

**Table 1:** Nucleotide sequence for primers used for qRT-PCR.

Gene	Forward Sequence (5′-3′)	Reverse Sequence (3′-5′)	Amplicon Length (bp)
18s	GGCCCGAAGCGTTTACTT	ACCTCTAGCGGCGCAATAC	173
CCR7	GCTCTCCTGGTCATTTTCCA	AAGCACACCGACTCATACAGG	107
CD163	TCATTTCGAAGAAGCCCAAG	CTCCGTGTTTCACTTCCACA	101
TNF-α	ACTCGAGTGACAAGCCCGTA	CCTTGTCCCTTGAAGAGAACC	184
MyoD	CGTGGCAGTGAGCACTACAG	TGTAGTAGGCGGCGTCGTA	133
eMHC	TGGAGGACCAAATATGAGACG	CACCATCAAGTCCTCCACCT	180
COL1	CTGGTGAACGTGGTGCAG	GACCAATGGGACCAGTCAGA	123

## Data Availability

The original contributions presented in this study are included in the article; further inquiries can be directed to the corresponding author.
